# Importation of Zika Virus from Vietnam to Japan, November 2016

**DOI:** 10.3201/eid2307.170519

**Published:** 2017-07

**Authors:** Takehiro Hashimoto, Satoshi Kutsuna, Shigeru Tajima, Eri Nakayama, Takahiro Maeki, Satoshi Taniguchi, Chang-Kweng Lim, Yuichi Katanami, Nozomi Takeshita, Kayoko Hayakawa, Yasuyuki Kato, Norio Ohmagari

**Affiliations:** National Center for Global Health and Medicine, Tokyo, Japan (T. Hashimoto, S. Kutsuna, Y. Katanami, N. Takeshita, K. Hayakawa, Y. Kato, N. Ohmagari);; National Institute of Infectious Diseases, Tokyo (S. Tajima, E. Nakayama, T. Maeki, S. Taniguchi, C.-K. Lim)

**Keywords:** Zika virus, viruses, Vietnam, Asia, Japan, infection, mosquitoes, vector-borne infections

## Abstract

We report a case of Zika virus infection that was imported to Japan by a traveler returning from Vietnam. We detected Zika virus RNA in the patient’s saliva, urine, and whole blood. In the Zika virus strain isolated from the urine, we found clearly smaller plaques than in previous strains.

Zika virus has been documented in Southeast Asia since the 1940s; however, the prevalence and geographic extent of Zika virus disease in Asia remain unclear (*1*). In Vietnam, 219 cases of Zika virus infection were reported in 2016 and 13 new cases in 2017 (*2*). We report a case of Zika virus infection imported from Vietnam to Japan, diagnosed after PCR amplification of Zika virus RNA in the patient’s saliva, urine, and whole blood.

A 40-year-old man came to the National Center for Global Health and Medicine in Tokyo, Japan, in the middle of November 2016 with fever and a rash. In early November 2016, he had traveled to Ho Chi Minh City, Vietnam, where he stayed for 10 days. During his return to Japan, he developed fever and a diffuse rash on his face, trunk, arms, and legs. He went to the hospital on the day after his return and reported having been bitten by mosquitoes in Ho Chi Minh City. Upon arrival at the hospital, he had no fever (temperature 36.8°C); physical examination revealed conjunctivitis and a maculopapular rash on his face, trunk, and extremities. Results of laboratory tests showed leukopenia (2,250 cells/μL; reference 3,500–8,500 cells/μL) and a platelet count within reference range. We performed a rapid dengue test (Dengue Duo NS-1 Ag + Ab combo; SD Bioline, Standard Diagnostics Inc., Gyeonggi-do, South Korea); results were negative for nonstructural protein 1, IgM, and IgG. We performed real-time reverse transcription PCR (RT-PCR) amplification using Zika virus primers and probes with urine, saliva, whole blood, serum (obtained 4 days after symptom onset), and semen samples (obtained 6 days after symptom onset). We detected Zika virus RNA in the urine (cycle threshold [C_t_] 32.0), saliva (C_t_ 39.1), and whole blood (C_t_ 38.1) samples. However, we did not detect Zika virus RNA in the serum or semen samples. We diagnosed Zika virus infection in this patient; his symptoms resolved without treatment within 7 days after he initially sought care. 

We successfully isolated the infectious Zika virus from the urine specimen. We amplified the nearly complete genome (10,696 bases; GenBank accession no. LC219720) of the Zika virus from the isolate using RT-PCR and subsequently sequenced it. BLAST analysis (https://blast.ncbi.nlm.nih.gov/Blast.cgi) showed that the isolate was an Asian lineage virus (*3*), sharing 99.3% sequence identity with the Zika virus strain isolated in French Polynesia in 2013 (H/PF/2013; GenBank accession no. KJ776791), 98.9% identity with the strain isolated in Fiji in 2016 (ZIKV/Hu/S36/Chiba/2016; GenBank accession no. LC191864), 99.0% identity with the strain isolated in Puerto Rico in 2015 (PRVABC59; GenBank accession no. KU501215), and 88.5% identity with the strain isolated in Uganda in 1947 (MR766-NIID; GenBank accession no. LC002520). The phylogenetic tree that we constructed using the complete coding region of the Zika virus genome suggested that the sequence belonged to the Southeast Asian clade of the Asian lineage (Figure). The plaque size of the isolated strain in Vero cells was obviously smaller than that of the Asian strain ZIKV/Hu/S36/Chiba/2016 (Pacific clade) (Technical Appendix).

**Figure:**
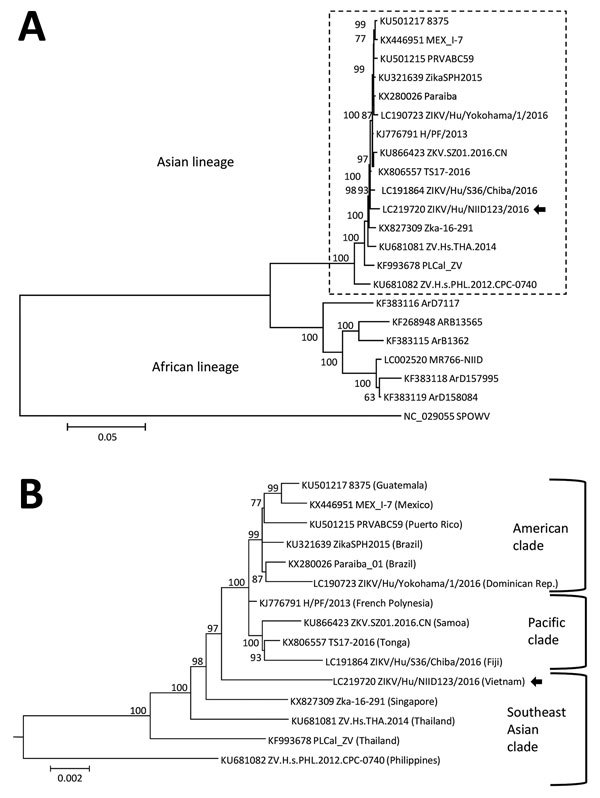
Phylogenetic analysis of the Zika virus sequence derived from a patient returning to Japan from Vietnam in November 2016. The phylogenetic tree was based on a nearly complete genome and constructed by using the maximum-likelihood method (MEGA 7.0, http://www.kumarlab.net/publications). The sequence derived from the patient is indicated with an arrow. A) The phylogenetic tree based on a nearly full-length region. B) The expanded Asian lineage branch (dotted box in panel A). Scale bars indicate nucleotide substitutions per site.

A previous study showed that Zika virus RNA could be detected more easily in urine than in serum a few days after disease onset (*4*). Other reports showed that Zika virus RNA could be detected for a longer period in whole blood than that in urine and serum (*5*) and that Zika virus RNA could be detected more easily in saliva than in plasma and urine during the first week after symptom onset (*6*). In the case we report, during RT-PCR analysis of the patient’s serum, urine, saliva, whole blood, and semen samples, the urine sample showed the lowest C_t_, indicating that the urine sample had the highest concentration of Zika virus RNA. However, we could not detect the Zika virus genome in serum and semen samples.

Our phylogenetic analysis suggested that the Asian lineage can be divided into 3 clades (Southeast Asian, Pacific, and American), and the strain we isolated belonged to the Southeast Asian clade. A previous study indicated that a strain isolated in Singapore in 2016 (ZKA-16-291; GenBank accession no. KX827309) also belonged to the Southeast Asian branch, and it was distinct from the isolates obtained in the Americas (*7*). Therefore, it is possible that the isolate from the current case was the strain that is already circulating in Vietnam and was not imported from South America. Our isolate formed smaller plaques in Vero cells than those observed with the other Asian lineage ZIKV/Hu/S36/Chiba/2016 strain (Pacific clade). We also confirmed that the plaque sizes of the PRVABC59 (American clade) and MR766-NIID (African lineage) strains resemble that of ZIKV/Hu/Chiba/S36/2016 (data not shown), suggesting that the Southeast Asian clade Zika virus strains might have a lower cytotoxicity and replicative ability than the American clade and African lineage.

In conclusion, the replicative ability of Zika virus might differ by region and thus influences endemic potential. Further studies are necessary to validate these findings.

Technical Appendix Comparison of plaque morphology between 2 Asian lineage strains of Zika virus, ZIKV/Hu/S36/Chiba/2016 and ZIKV/Hu/NIID123/2016.

## References

[1] Meltzer E, Lustig Y, Leshem E, Levy R, Gottesman G, Weissmann R, et al. Zika virus disease in traveler returning from Vietnam to Israel. Emerg Infect Dis. 2016;22:1521–2. 10.3201/eid2208.160480 27331627 PMC4982162

[2] Zika virus (02): Americas, Asia, Africa, Pacific, research, observations. ProMed. 2017 Feb 17 [cited 2017 Mar 20]. https://www.promedmail.org/post/4846633, archive no. 20170217.4846633.

[3] Haddow AD, Schuh AJ, Yasuda CY, Kasper MR, Heang V, Huy R, et al. Genetic characterization of Zika virus strains: geographic expansion of the Asian lineage. PLoS Negl Trop Dis. 2012;6:e1477. 10.1371/journal.pntd.0001477 22389730 PMC3289602

[4] Bingham AM, Cone M, Mock V, Heberlein-Larson L, Stanek D, Blackmore C, et al. Comparison of test results for Zika virus RNA in urine, serum, and saliva specimens from persons with travel-associated Zika virus disease—Florida, 2016. MMWR Morb Mortal Wkly Rep. 2016;65:475–8. 10.15585/mmwr.mm6518e2 27171533

[5] Lustig Y, Mendelson E, Paran N, Melamed S, Schwartz E. Detection of Zika virus RNA in whole blood of imported Zika virus disease cases up to 2 months after symptom onset, Israel, December 2015 to April 2016. Euro Surveill. 2016;21:30269. 10.2807/1560-7917.ES.2016.21.26.30269 27386894

[6] Barzon L, Pacenti M, Berto A, Sinigaglia A, Franchin E, Lavezzo E, et al. Isolation of infectious Zika virus from saliva and prolonged viral RNA shedding in a traveller returning from the Dominican Republic to Italy, January 2016. Euro Surveill. 2016;21:30159. 10.2807/1560-7917.ES.2016.21.10.30159 26987769

[7] Maurer-Stroh S, Mak TM, Ng YK, Phuah SP, Huber RG, Marzinek JK, et al. South-east Asian Zika virus strain linked to cluster of cases in Singapore, August 2016. Euro Surveill. 2016;21:30347. 10.2807/1560-7917.ES.2016.21.38.30347 27684526 PMC5073200

