# Supplementary material for: Importation of Zika Virus from Vietnam to Japan, November 2016
Source: Emerg Infect Dis. 2017 Jul;23(7):1223–5. doi: 10.3201/eid2307.170519 (PMC5512472; doi:10.3201/eid2307.170519)
Supplement: Technical Appendix — Comparison of plaque morphology between 2 Asian lineage strains of Zika virus, ZIKV/Hu/S36/Chiba/2016 and ZIKV/Hu/NIID123/2016. [file 17-0519-Techapp-s1.pdf]

Article DOI: https://dx.doi.org/10.3201/eid2307.170519

Importation of Zika Virus from Vietnam to Japan, November 2016

Technical Appendix

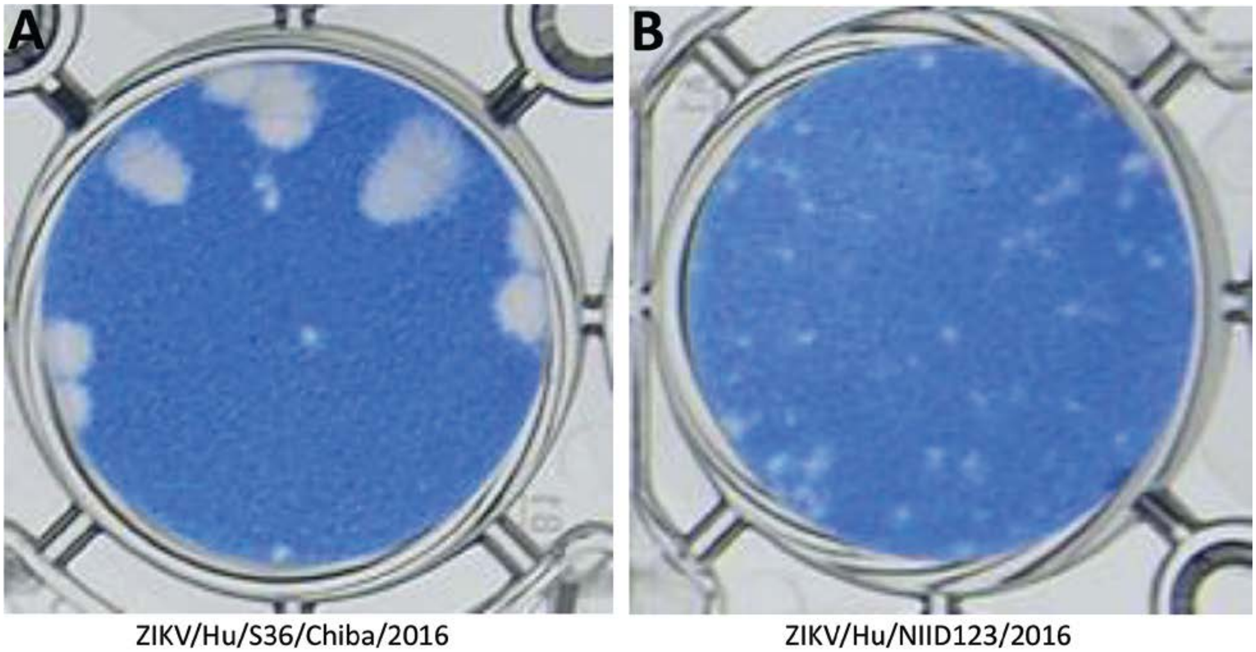

Technical Appendix Figure. Comparison of plaque morphology between 2 Asian lineage strains of Zika virus. A) ZIKV/Hu/S36/Chiba/2016. B) ZIKV/Hu/NIID123/2016.
